# Robotic-Assisted Total Knee Arthroplasty: Current Evidence on PROMs, Functional Outcomes, Neuromotor Recovery, and Complications—A Narrative Review

**DOI:** 10.3390/medicina62061173

**Published:** 2026-06-17

**Authors:** Bogdan-Sorin Capitanu, Serban Dragosloveanu, Dana-Georgiana Nedelea, Calin Ion Dragosloveanu, Romica Cergan, Cristian Scheau

**Affiliations:** 1 Faculty of Medicine, The “Carol Davila” University of Medicine and Pharmacy, 050474 Bucharest, Romania; 2 Department of Orthopaedics, “Foisor” Clinical Hospital of Orthopaedics, Traumatology and Osteoarticular TB, 021382 Bucharest, Romania; 3 Department of Radiology and Medical Imaging, “Foisor” Clinical Hospital of Orthopaedics, Traumatology and Osteoarticular TB, 021382 Bucharest, Romania

**Keywords:** robotic-assisted total knee arthroplasty, functional outcomes, neuromotor recovery, gait analysis, proprioception, Physiology, patient satisfaction

## Abstract

*Background and Objectives*: Robotic-assisted total knee arthroplasty (rTKA) is being increasingly used to improve surgical precision, soft-tissue balancing, and functional recovery. However, evidence comparing rTKA with conventional manual TKA (mTKA) across functional, patient-reported, neuromotor, and safety outcomes remains heterogeneous. *Materials and Methods*: This narrative (non-systematic) review synthesises studies evaluating functional outcomes, patient-reported outcome measures (PROMs), joint awareness, range of motion (ROM), neuromotor recovery, and complications following rTKA versus mTKA. Study inclusion was based on author judgement and data accessibility. The reviewed evidence included five randomised controlled trials, 9 retrospective studies, six prospective non-randomised studies, two meta-analyses, one cross-sectional study, and one umbrella review, covering CT-based and imageless robotic platforms, including semi-active and active systems such as MAKO, NAVIO, CORI, ROSA, ROBODOC, CUVIS Joint, SkyWalker, TSolution One, AKEC, JIANJIA, and YUANHUA. *Results*: rTKA consistently demonstrated outcomes comparable to mTKA in PROMs (OKS, KOOS, WOMAC, KSS), with some studies reporting modest early improvements in pain and function. Joint awareness and patient satisfaction showed the most consistent early advantages for rTKA. Early postoperative ROM and neuromotor recovery, including balance and gait symmetry, were improved with rTKA, likely due to enhanced alignment and soft-tissue balancing; however, mid- and long-term outcomes were similar. Complication rates were low and comparable, with robotic-specific issues being rare and self-limited. *Conclusions*: rTKA provides small but reproducible early benefits in joint awareness, neuromotor function, and patient satisfaction, without clear long-term superiority. These early advantages may translate into meaningful population-level benefits, including faster recovery and potential healthcare cost reduction. Further high-quality studies are needed to assess long-term clinical and economic outcomes.

## 1. Introduction

Knee osteoarthritis is a progressive condition frequently requiring total knee arthroplasty (TKA) in advanced stages [1,2,3]. Despite continuous improvements in implant design and surgical techniques patient dissatisfaction after conventional manual TKA remains in the range of 20–25%, whereas satisfaction after conventional manual total hip arthroplasty commonly approaches 90% [4,5,6,7].

A major source of dissatisfaction is the absence of a “natural knee” sensation after surgery [8]. This phenomenon is largely related to alterations in knee kinematics, soft-tissue balance, and load distribution following implantation, which may affect proprioception, stability, and overall joint perception [9,10]. The most widely adopted strategy, mechanical alignment (MA), seeks to restore a neutral limb axis using cuts perpendicular to the mechanical axes of the femur and tibia [11]. Mechanical alignment is favoured for its reproducibility, symmetric load distribution across compartments, and implant longevity. However, it does not account for the fact that only a minority of patients exhibit truly neutral native alignment [12,13,14,15]. In response, kinematic alignment (KA) was developed to better replicate each patient’s native anatomy, with the intention of improving functional outcomes and joint perception. More recently, alignment philosophies have evolved further toward concepts such as restricted kinematic, inverse kinematic, and functional alignment, all pursuing the same objective: recreation of patient-specific (native) joint kinematics [16,17]. Building on these principles, newer approaches have extended beyond alignment alone toward individualised component positioning, integrating patient-specific anatomy with soft-tissue balance and the constraints imposed by implant design to optimise overall knee function [18,19]. In routine clinical practice, though, executing precise, patient-specific bone resections and reproducing them consistently is challenging with manual instruments alone and is more reliable with patient-specific instrumentation or robotic assistance [20,21,22].

These limitations have contributed to the growing interest in technologies aimed at improving surgical accuracy, reproducibility, and soft-tissue preservation, with the ultimate goal of improving the patient experience. Robotic-assisted TKA (rTKA) appeared in the early 2000s and has progressed significantly in the following years [23]. Contemporary systems provide pre- or intraoperative mapping and constrained, semi-active (haptic) guidance designed to reduce outliers in component position and limb alignment, as well as to optimise soft-tissue balance within patient-specific alignment targets, rather than enforcing a uniform mechanical alignment [24,25]. The proposed pathway to clinical benefit is therefore not simply related to achieving neutral alignment, but to improving the consistency with which planned, individualised alignment and ligament balance are reproduced intraoperatively. In this context, robotic platforms may also facilitate the implementation of patient-specific alignment strategies, such as kinematic alignment and its derivatives, potentially enhancing the restoration of native joint mechanics. Emerging evidence suggests that reproducing coronal alignment phenotypes, particularly in patients with constitutional varus alignment (e.g., CPAK type I), may be associated with improved functional outcomes and patient satisfaction when compared to systematic correction to mechanical neutrality. However, this approach remains dependent on appropriate safety boundaries and patient selection, and current evidence is still heterogeneous [26,27,28]. When bone resections and implant positioning more closely match patient specific targets, abnormal contact pressures, instability, and maltracking may be reduced [29,30]. This, in turn, may facilitate earlier pain relief, smoother early recovery, and better functional outcomes and satisfaction. Beyond pain and range of motion (ROM), more accurate reproduction of patient-specific alignment and soft-tissue balance may also influence neuromotor variables, such as proprioception, gait symmetry, and muscle activation. Although long-term data remain limited, early and mid-term findings are encouraging [31,32].

Despite these theoretical advantages, the clinical translation of robotic accuracy remains uncertain. Recent randomised evidence suggests that rTKA improves radiological accuracy and reduces mechanical-axis outliers compared with conventional TKA, but these improvements have not yet consistently resulted in clinically meaningful differences in PROMs or ROM. This discrepancy highlights the need to evaluate rTKA beyond alignment accuracy alone and to consider broader outcome domains, including patient perception, functional recovery, neuromotor performance, and complications [33].

This narrative review examines four major outcome domains commonly used to evaluate results after TKA: patient-reported outcome measures (PROMs), functional outcomes, neuromotor recovery, and complications. These domains were selected to provide a comprehensive and multidimensional assessment of postoperative performance, including both patient-centred outcomes and objective clinical measures.

Although these outcome domains are widely used in contemporary research, they are frequently evaluated separately, with most studies focusing on a limited subset of parameters. However, postoperative recovery and overall patient satisfaction are influenced by the complex interaction between multiple factors rather than a single domain alone.

**Figure 1:**
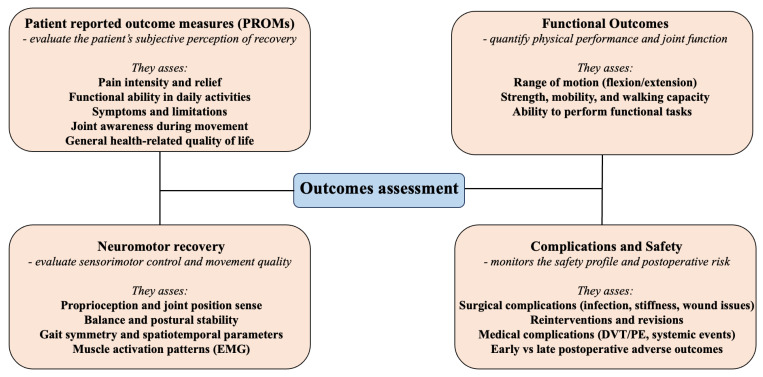
Key outcome domains commonly used to assess recovery after TKA.

Therefore, in addition to summarising the available evidence, this review aims to highlight the fragmentation within the current literature and to emphasise the need for a more integrated and multidimensional approach when assessing outcomes following TKA. The schematic diagram below (Figure 1) illustrates the four outcome domains included in this review and the key aspects they are commonly used to assess.

## 2. Materials and Methods

This review was structured around the following guiding questions: (1) Does robotic assistance improve functional outcomes after primary TKA compared with conventional manual techniques at early (≤90 days), mid-term (3–12 months), and later (>12 months) time points? (2) Does rTKA affect patient satisfaction, including global scores? (3) Are there measurable differences in neuromotor recovery (proprioception, balance, gait, EMG) attributable to rTKA?

A literature search was conducted in the major medical databases (PubMed/MEDLINE, Embase, Cochrane Library, and Scopus) to identify relevant studies published in English. Search terms included combinations related to robotic-assisted knee arthroplasty and postoperative outcomes (e.g., “robotic knee arthroplasty”, “robot-assisted total knee replacement”, “functional outcomes”, “patient satisfaction” “forgotten joint score”, “gait”, “balance”, “proprioception”, “electromyography”, “complications”). Additional sources were identified through manual screening of reference lists from key publications and recent reviews. Because study selection relied partly on author judgement and availability of full-text data, the included evidence reflects a selected synthesis rather than a comprehensive, protocol-driven search.

The reviewed evidence included 5 randomised controlled trials, 9 retrospective studies, 6 prospective non-randomised studies, 2 meta-analyses, 1 cross-sectional study, and 1 umbrella review and covered CT-based and imageless robotic platforms, including semi-active and active systems, such as MAKO SmartRobotics™ (Stryker Corporation, Portage, MI, USA), NAVIO™ Surgical System (Blue Belt Technologies, Inc., a Smith+Nephew company, Plymouth, MN, USA), CORI™ Surgical System (Blue Belt Technologies, Inc., a Smith+Nephew company, Pittsburgh, PA, USA), ROSA^®^ Knee System (Zimmer Biomet, Warsaw, IN, USA), DigiMatch ROBODOC^®^ Surgical System (Integrated Surgical Systems, Inc., Sacramento, CA, USA), CUVIS-joint (CUREXO, Inc., Seoul, Republic of Korea), SkyWalker^®^ Surgical Robot (MicroPort NaviBot (Suzhou) Co., Ltd., Suzhou, China), TSolution One^®^ Total Knee Application (THINK Surgical, Inc., Fremont, CA, USA), AKEC/K3 Orthopedic Robotic System (AK Medical, Beijing, China), ARTHROBOT Knee/JIANJIA robotic system (Hangzhou Jianjia Medical Technology Co., Ltd., Hangzhou, China), and KUNWU^®^/YUANHUA Robotic Orthopaedic Surgical System (Yuanhua Orthopaedic Robotics (Shenzhen) Limited, Shenzhen, China).

Eligibility criteria included studies reporting patients undergoing primary TKA that (a) directly compared rTKA with mTKA or (b) reported outcomes after rTKA within at least one of the four domains of interest. Randomised trials, prospective and retrospective cohorts, registry analyses, and relevant biomechanical/physiologic studies were considered. Exclusion criteria comprised unicompartmental knee arthroplasty, revision procedures, cadaveric studies without clinical outcomes, case reports, and conference abstracts without full data.

Data items of interest included study design, sample size, rehabilitation context, follow-up duration, and outcomes: PROMs (OKS, KOOS, WOMAC, KSS, FJS-12 and EQ-5D), objective function (ROM, TUG, 6MWT, gait speed), satisfaction measures, neuromotor metrics (proprioception tests, balance/posturography, gait symmetry, EMG), complications/readmissions, and, where available, minimal clinically important differences (MCIDs).

MCID represents the smallest change in an outcome measure that is perceived as beneficial by the patient and provides a clinically meaningful interpretation beyond statistical significance [34]. Where available, reported outcomes were interpreted in the context of established MCID thresholds to distinguish between statistically significant differences and those likely to be clinically relevant. However, it should be noted that MCID thresholds are instrument-specific and may vary across patient populations, and no universal consensus exists for defining clinically meaningful or poor outcomes after TKA [35,36].

For a comprehensive understanding, particularly for readers who may be less familiar with these outcome measures, a brief overview of the PROMs included in this review, together with their clinical use, is provided in the Appendix A section of this paper.

## 3. Technology and Surgical Concepts

Robotic technology traces back to 1988 in neurosurgery, with ROBODOC^®^ subsequently becoming the first robot used in orthopaedics, initially for hip arthroplasty and later adapted to TKA [37]. Since then, the field has diversified into platforms with distinct operating principles. By execution level, systems are commonly classified as passive, semi-active, or active. Passive systems provide intraoperative guidance (e.g., alignment, orientation, gap assessment) but do not execute bone cuts, accuracy, and reproducibility, therefore remaining largely technique dependent. Semi-active systems add robotic constraints, where the surgeon moves the instrument while the robot enforces virtual boundaries (haptics) or positions cutting guides with high precision, improving execution while maintaining surgeon control. Active systems autonomously perform bone preparation according to the operative plan under surgeon supervision, offering high geometric fidelity at the cost of greater complexity and capital requirements [38].

Workflows also differ by imaging strategy. Image-based approaches use preoperative CT, MRI, or calibrated radiographs to build a patient-specific 3D model and preplan implant size, position, and alignment, and this plan is then registered intraoperatively and executed with navigation or haptic guidance [39]. This provides excellent anatomic fidelity, particularly valuable in severe deformity, extra-articular malalignment, or retained hardware, while introducing imaging logistics, costs, and a small radiation dose. Imageless robots exclude preoperative imaging, creating the plan intraoperatively by digitising bony landmarks and limb kinematics with optical or inertial trackers. They avoid radiation and simplify the preoperative process, but their accuracy depends on consistent landmarking and assumptions regarding cartilage wear [40,41].

With respect to alignment philosophy, contemporary practice is usefully framed as mechanical alignment versus kinematic alignment and its variants. Kinematic alignment, including restricted and inverse approaches, aim to reproduce patient-specific joint lines and laxity within predefined safety bounds [42,43]. More recent concepts further integrate alignment strategies with individualised component positioning, acknowledging both patient-specific anatomy and implant-related constraints [18,19]. Modern robotic platforms can implement either strategy, selection should be guided by case complexity, institutional resources, and workflow priorities, balancing precision and reproducibility against cost and throughput [43].

## 4. Patient-Reported Outcomes (PROMs)

PROMs are standardised, patient-completed questionnaires that capture functional status, general health, and condition-specific outcomes. They reduce clinician–assessor bias and provide a patient-centred measure of health status [44]. In this section, we consider studies reporting generic PROMs (e.g., SF-36, EQ-5D) and condition-specific PROMs (e.g., OKS, KOOS, KOOS-JR WOMAC, KSS, FJS-12) after rTKA compared with mTKA technique. Table 1 summarises the results.

PROMs capture multiple dimensions of postoperative recovery, including pain, function, joint awareness, and quality of life, which may contribute to variability in reported outcomes. Condition-specific PROMs, such as OKS, KOOS/KOOS-JR, WOMAC, and KSS, primarily capture knee pain and basic functional capacity. These domains tend to improve in both rTKA and mTKA during the first postoperative months, and many studies including Geng et al. [45], Naziri et al. [46], Albelooshi et al. [49], Mitchell et al. [50], and Held et al. [53] report no significant between-group differences at early or 1–2-year follow-up. This pattern reflects the well-known ceiling effect of these scales, defined as a limitation whereby patients achieve high scores postoperatively, leaving little room for further measurable improvement despite potential clinical differences; once postoperative pain and disability fall below a certain threshold, small improvements derived from surgical technique become harder to detect [56,57,58].

Nevertheless, some studies show early functional advantages for rTKA. Han et al. [48] and Golinelli et al. [47] found better PROMs at the 6-month interval (with Golinelli et al. showing superiority in most measures except KOOS-PS). These findings suggest that robotic assistance may accelerate early symptom relief or functional gains, although the effect is inconsistent and diminishes over time.

Another evaluated PROM is the FJS-12, which measures joint awareness. Findings remain inconsistent: Eerens et al. [55] reported higher FJS-12 scores after rTKA while others showed no difference (Stoltz et al. [54]) or even favoured mTKA (Yamamoto et al. [51]). This inconsistency suggests that reductions in joint awareness may depend more on alignment philosophy, implant design, soft-tissue balancing, and patient expectations than on robotic assistance alone. Nevertheless, FJS-12 may be one of the PROMs with greater potential to detect differences between rTKA and mTKA, as it evaluates joint awareness and has a lower ceiling effect than several traditional PROMs. In this context, Zhang et al., in a recent systematic review and meta-analysis focused specifically on joint awareness after joint arthroplasty, reported that rTKA achieved higher FJS-12 scores than manual TKA across short-, mid-, and long-term follow-up periods [59]. Therefore, while the findings of the studies included in the present review remain heterogeneous, the broader literature suggests that FJS-12 may be particularly relevant for detecting patient-perceived differences after rTKA [60].

Generic health-related QoL instruments (EQ-5D, SF-12/36, VR-12) are even less capable of detecting subtle differences between rTKA and mTKA. Except for Golinelli et al. [47], who reported improved EQ-VAS and EQ-5D at 6 months in the rTKA group, most studies found no significant differences. These results suggest that although robotic precision in alignment and gap balancing is superior to conventional techniques, it does not necessarily translate into improvements in broader physical or emotional health domains, which are often influenced by factors such as comorbidities, social context, and rehabilitation quality.

Patient satisfaction, evaluated in several studies, generally reflects the findings from PROMs but provides additional information. Stoltz et al. [54] reported higher satisfaction rates after rTKA, including statistically significant improvements, despite heterogeneous PROM findings between groups. This may suggest that there are some aspects of patient experience that are not fully captured by standard PROMs. In contrast, Liow et al. [52] found no difference in satisfaction despite better SF-36 vitality and emotional role components in the robotic group, again suggesting the complex interplay between surgical technique and subjective perception.

Overall, PROMs and satisfaction evidence indicate that rTKA consistently performs at least as well as mTKA and may offer early advantages in pain, function, and patient satisfaction in select cohorts. The results supporting robotic-assisted surgery come primarily from FJS-12 scores and patient satisfaction, but the findings remain heterogeneous. These results suggest that while enhanced accuracy and reproducibility are necessary, they are not sufficient alone. Patient-perceived outcomes may also depend on alignment philosophy, implant design, perioperative care, and rehabilitation pathways [6,61,62,63].

## 5. Functional Outcomes

Functional outcomes after TKA describe a patient’s measurable physical performance, including mobility, joint motion, and the ability to perform daily activities [64]. Although many studies assess functional recovery using various objective tests, the only consistently reported and comparable metric across the rTKA literature is postoperative range of motion (ROM). For this reason, ROM was the primary functional outcome assessed in this review, as it offers the most reliable and uniform data for evaluating differences between rTKA and mTKA.

ROM represents the total angular movement of the knee joint, usually from maximum extension to maximum flexion, and is one of the main indicators of functional recovery and joint mobility [65]. rTKA improves the accuracy of component placement, joint-line restoration, and individualised soft-tissue balancing, factors which are considered to positively influence patient outcomes, including ROM, and may contribute to faster early postoperative recovery [29,66]. This section summarises the findings from studies reporting these measures (Table 2).

Postoperative ROM is a key determinant of functional recovery after TKA, and numerous studies have assessed whether rTKA offers measurable advantages over mTKA. Overall, the evidence suggests that robotic assistance may provide modest early benefits, although long-term ROM appears equivalent between techniques. From a clinical perspective, flexion values in the range of approximately 110–120° are generally considered sufficient for most activities of daily living and are traditionally regarded as satisfactory outcomes [71,72]. However, these reference values are not universally standardised, and no consensus exists defining a strict threshold for “good” or “poor” ROM after TKA. Reported outcomes vary depending on patient population, baseline characteristics, surgical technique, and follow-up duration [35,36]. Therefore, in the present review, ROM findings were interpreted in a comparative context between rTKA and conventional techniques, rather than against a predefined absolute standard.

Early postoperative advantages are reported in several studies. Han et al. [48] observed greater improvement in ROM at six months after rTKA, and Fary et al. [68] demonstrated less early motion loss at one month and a faster return to 90° of flexion by three months. Additional early gains were noted by Naziri et al. [46], who reported significantly improved ROM at 90 days in the robotic group. These findings support the hypothesis that improved implant alignment, more accurate joint-line restoration, and precise soft-tissue balancing reduce early postoperative stiffness, inflammation and pain and facilitate more efficient rehabilitation. However, a larger number of studies (Major et al. [67], Liow et al. [52], Tian et al. [69], Xu et al. [70], and Held et al. [53]) found no significant differences between rTKA and mTKA at early or late follow-up intervals, including 1–2-year assessments. Even among studies showing slightly better values in the robotic group, the amount of improvement was small. Yamamoto et al. [51] found slightly better extension in rTKA, though neither were clinically nor statistically significant. Similarly, Stoltz et al. [54] observed a statistically significant difference in flexion favouring rTKA at two years, but the absolute difference was not clinically meaningful, and extension was comparable.

Overall, the current evidence indicates that rTKA may enhance early ROM recovery, but in mid- to long-term follow-up, ROM outcomes converge between robotic and conventional techniques. Early postoperative ROM is strongly influenced by pain, swelling, muscle inhibition, and patient engagement in physiotherapy, factors that may improve faster following rTKA due to reduced soft-tissue trauma, optimised balancing, or more predictable kinematics [73,74]. In this context, robotic assistance should be viewed not only as a tool that improves accuracy and reproducibility but also as a platform that facilitates the implementation of specific alignment strategies. By enabling more precise adaptation to patient-specific alignment philosophies, robotic systems may contribute to restoring joint mechanics and kinematics closer to the native state, which could positively influence early ROM recovery [74]. Nevertheless, postoperative ROM remains a multifactorial outcome, influenced by additional variables such as implant design, component positioning, surgical technique and approach, patient-specific characteristics, and perioperative factors, including anticoagulation protocols [75]. While the early gains may support faster functional progression and gait normalisation, their long-term clinical relevance remains uncertain.

## 6. Neuromotor Recovery

Neuromotor recovery following TKA represents a critical component of postoperative functional restoration, including the re-establishment of coordinated interactions between sensory input, central motor control, and muscular output. Beyond structural alignment and pain reduction, the return of efficient neuromotor function, defined by proprioceptive acuity, quadriceps activation, postural stability, and gait symmetry, determines the quality and naturalness of movement after surgery [76,77]. By minimising arthrogenic muscle inhibition, improving load distribution, and reducing aberrant joint mechanics, robotic assistance may support faster normalisation of neuromuscular control and improve functional outcomes [78,79]. Currently, there is no universally accepted or standardised outcome measure for assessing neuromotor recovery after TKA, with studies relying on a wide range of assessment tools. Table 3 summarises the main findings from studies evaluating neuromotor recovery after rTKA.

The clinical interpretation of neuromotor outcomes remains challenging because most available measures are reported as statistically significant differences rather than in relation to validated minimal clinically important differences (MCIDs). In the available TKA literature, isokinetic dynamometry provides an objective assessment of quadriceps and hamstring performance, while surface electromyography allows evaluation of muscle activation patterns. However, clinically meaningful thresholds for these measures are not consistently reported in TKA cohorts [83]. Similarly, the Berg Balance Scale is a clinically familiar tool for postural control, but its interpretation in rTKA studies is limited by small sample sizes and the absence of predefined TKA-specific MCID interpretation [84,85]. Advanced gait analysis provides sensitive spatiotemporal, kinetic, and kinematic data, but standardised MCIDs for many gait-derived variables after TKA are not consistently established [86]. Therefore, neuromotor findings should be interpreted cautiously, with a focus on consistency across related domains rather than isolated statistically significant findings alone.

Neuromotor recovery after TKA reflects the integration of proprioception, dynamic balance, muscle control, and gait symmetry. Across available studies, rTKA shows improvements of earlier functional reactivation compared with mTKA, although long-term neuromotor outcomes are mostly similar between techniques.

Early postoperative findings suggest that relevant neuromuscular deficits persist despite robotic precision. García-Sanz et al. evaluated quadriceps activation using surface electromyography four weeks after robotic-assisted TKA and found lower vastus medialis and vastus lateralis activation on the operated limb compared with the non-operated limb, together with reduced sagittal-plane ROM during gait in female patients. These findings indicate that arthrogenic muscle inhibition and limb asymmetry remain present during the early postoperative period, even after robot-assisted implantation [74].

Mid-term data, such as that reported by Han et al., show small improvements in isokinetic quadriceps strength after rTKA at around 6 months; however, the reported differences were not interpreted using validated MCID thresholds for isokinetic performance, and therefore their clinical magnitude should be considered with caution [48].

Regarding mid- to long-term results, Bayrak et al. found significantly better performance on the Berg Balance Scale in the rTKA group, indicating improved postural control and proprioceptive stability, although other functional and patient-reported measures, including Lysholm score, SF-12, FJS-12, pain during activity, and satisfaction, were not significantly different between groups. This suggests that rTKA may provide some advantage in postural control, but this isolated balance improvement does not necessarily translate into broader functional superiority [82].

Ajekigbe et al. demonstrated more efficient gait mechanics in the rTKA group with reduced lateral sway and improved propulsion timing, suggesting smoother neuromotor coordination and improved trunk–lower limb synergy [81]. However, no broad superiority over mTKA was observed across most gait parameters or plantar pressure distribution. Advanced gait analysis provides further insight. He et al. observed notable improvements at 3 and 6 months in the rTKA group, including reduced double-support time and increased knee ROM during gait cycles. Nevertheless, mild residual asymmetries compared with the contralateral limb persisted, including reduced external rotation and higher joint moments, suggesting that neuromotor restoration remains an extended process even with robotic precision [80].

Taken together, the evidence indicates that robotic assistance may provide more stable early balance, more symmetrical early gait patterns, and potentially faster proprioceptive adaptation, likely due to improved alignment, joint-line restoration, and consistent soft-tissue tensioning. However, these effects are heterogeneous, often modest, and not consistently supported by MCID-based interpretation. Quadriceps inhibition, gait asymmetry, and neuromuscular deficits still occur and may persist beyond the early recovery phase. By mid- to long-term follow-up, neuromotor metrics, including gait parameters and isokinetic strength, appear to equalise between rTKA and mTKA. Robotic precision seems to contribute to selected aspects of earlier neuromotor recovery, but full normalisation still depends on rehabilitation quality and patient-specific factors [77].

## 7. Complications

TKA is a major surgical procedure associated with a range of potential complications, from minor to severe [87]. Over time, the evolution of surgical techniques, perioperative protocols, and prophylactic strategies has significantly reduced many of these risks, including infection, thromboembolic events, and postoperative stiffness [88]. The introduction of rTKA, similar to other robotic applications in surgery, aims to make the procedure less invasive, more precise, and less aggressive to soft tissues, thereby improving clinical outcomes and reducing complication rates or hospital stays [23,29,89].

However, despite these advantages, robotic-assisted surgery introduces its own potential risks, including pin site infections, periarticular fractures related to fixation pin placement or technical malfunctions [90,91,92,93]. Fortunately, these events remain relatively rare and are often outweighed by the benefits of enhanced precision and early rehabilitation. Table 4 summarises the key findings from studies reporting complications after rTKA.

Across the studies, the complication profile of rTKA was generally comparable to that of mTKA, with no consistent evidence of increased risk. The majority of investigations (Held et al. [53], Song et al. [97], Mitchell et al. [50], and Stoltz et al. [54]) report similar complication rates between the two techniques over follow-up periods ranging from 30 days to more than three years. These findings strengthen the overall safety of robotic-assisted procedures.

A small number of studies report complications specifically associated with robotic instrumentation. Liow et al. identified two reinterventions in the rTKA cohort with a periprosthetic joint infection and a tibial malposition causing persistent lateral knee pain, which both required surgical management [52]. Held et al. reported a non-displaced tibial shaft fracture attributed to a pin site, drawing attention to the known but uncommon risk related to tracker pin placement [53]. Kayani et al. similarly described a case of superficial wound dehiscence at a pin site, managed successfully with dressings and prophylactic antibiotics [94]. These pin site complications remain infrequent and typically resolve without major consequences.

Other studies report the absence of robotic-specific adverse events. Mahure et al. reported no complications attributable to robotic assistance in a cohort of 115 rTKA patients [96]. Short-term analyses, such as Naziri et al., found only one arthrofibrosis event requiring manipulation under anaesthesia, but only in the mTKA group [46]. Similarly, Smith et al. observed comparable numbers of complications in both groups, with equal rates of manipulation under anaesthesia [95].

Revision procedures are rare in both techniques. Albelooshi et al. documented a single periprosthetic femoral fracture requiring revision in the rTKA group, while long-term studies did not show increased revision risk associated with robotics [49].

Overall, complications after rTKA are low and comparable to mTKA, with pin-related events being the only reproducible robotic-specific issue, occurring at a low rate and being generally manageable without long-term sequelae. The current evidence suggests that robotic assistance does not increase overall complication rates and may, in some settings, reduce technical errors, such as malalignment-induced symptoms. Larger multicentre datasets with longer follow-up are needed to determine whether the improved accuracy associated with rTKA eventually translates into reduced revision rates over time.

## 8. Clinical Meaningfulness and Benchmark-Oriented Interpretation of Higher Informative Studies

Evaluation of rTKA results should go beyond comparison with average mTKA performance. A statistically non-significant result does not necessarily reflect failure if the absolute outcome in both cohorts is excellent. Likewise, a statistically significant difference may have no clinical relevance if the absolute result is poor or not what would be expected of quality TKA care. Therefore, the question is not only whether rTKA performs better than mTKA but if patients treated with rTKA will achieve excellent, benchmark-quality outcomes?

This goal fits with the achievable benchmarking framework, in which improvement efforts should be aimed at the best achievable outcomes (not averages or medians) in the real world; benchmarks in surgery should be excellent, reproducible, objective, and achievable based on robust and not arbitrary thresholds [98]. In TKA, this would translate to optimal quality in terms of patient satisfaction, joint awareness, functional ROM, manipulation under anaesthesia, readmission rate, infection rate, revision rate, and robotic-specific complications [99].

For this reason, not all studies included in this narrative review are appropriate for benchmarking. Studies were retained in the overall synthesis when they contributed relevant comparative information, but benchmark-oriented discussion is focused primarily on studies that reported preoperative or baseline values, absolute postoperative results, MCIDs or clinically meaningful improvement where available, satisfaction rates, ROM recovery, manipulation under anaesthesia, readmissions, complications, or revision. Studies with insufficient preoperative data or only limited postoperative comparison were interpreted with care and were not solely used to define the benchmark-level standard of care. This avoids outcome-based exclusion but maintains that poor or poorly reported results are not suitable for creating benchmarks.

Among PROM studies, one of the strongest benchmark-oriented articles comes from Golinelli et al. [47], in which the authors used pre-op and 6-month postoperative PROMs and reported on what proportion of patients achieved MCID improvement—68.9% of patients achieved MCID improvement for EQ-VAS, 58.3% for EQ-5D-3L, and 68.9% for KOOS-PS—while rTKA patients also demonstrated shorter length of stay and greater engagement in rehabilitation. This is a better method of reporting for benchmarking than just a statistical comparison between group means, as it measures what proportion of patients achieved a clinically significant outcome level.

Satisfaction is also likely a reliable benchmark-oriented outcome, as it measures overall patient perception; Smith et al. [95] found 94% rTKA patients were satisfied or very satisfied after 1 year, compared to 82% with mTKA, while Stoltz et al. [54] found similar 95.0% satisfaction with rTKA vs. 87.4% mTKA with mean follow-up >2 years. Again, it is reasonable to assume that patient satisfaction at these levels would be a more clinical measure than the difference in traditional PROM scores, especially in conjunction with a low rate of complication and revision. However, patient satisfaction is not an isolated measure and will likely be influenced by other factors as well, such as expectations and the experience of the surgery, implant design, and patient selection.

Joint awareness may also be a target for benchmark-oriented evaluation, but evidence here remains mixed; Eerens et al. [55] found higher FJS-12 and OKS with no change in adverse events or readmission with rTKA, while Stoltz et al. [54] found no difference in FJS-12 with better KSS, WOMAC, KOOS-JR, and satisfaction following robotic surgery—overall FJS-12 values were ~70 in both groups. Yamamoto et al. [51] further demonstrates that improvement in surgical accuracy does not always equate to patient outcomes, as while rTKA significantly improved rotational mismatch, it had a higher FJS-12 than the conventional surgery group. These values may be better measured with absolute postoperative outcomes against benchmarks rather than statistical comparison alone.

Range of motion offers an objective measure of function; however, its interpretation as a benchmark is challenging due to a lack of universally defined thresholds [35]. Nevertheless, data such as Fary et al.’s [68] study showing fewer early losses of active range of motion and a more rapid attainment of 90 degrees of flexion in rTKA without increasing adverse events, as well as data [46] showing better 90-day range of motion restoration with shorter length of stay and no difference in KSS, LEAS, patient satisfaction, or complication rates, provides a helpful benchmark-related outcome measure; meanwhile, the study by Major et al. [67] reporting no early flexion or extension difference and similar rates of manipulation under anaesthesia (3.0% vs. 2.5%) also highlights areas where no benchmark advantage may be achieved. Thus, range of motion should also be looked at with baseline data and, ideally, rate of restoration to 90 degrees, patient acceptable threshold, and necessity for manipulation under anaesthesia.

Complications provide a prime target for benchmark-oriented evaluation since their relevance is undeniable and their measurement largely objective: in the reviewed literature, robotic TKA did not show a greater rate of complications than conventional surgery. Naziri et al. [46] demonstrated comparable rates of major and minor complications through 90 days, while Kayani et al. [94] demonstrated a seven-case learning curve with no associated increase in implant positioning accuracy or complications, and Mahure et al. [96] demonstrated similar no learning curve-associated complications after active robotic TKA implantation. Thus, the general TKA complication rates were no higher for robotic surgeries, though robotic-specific complications, such as pin site infection, tracker fracture, retained material, or procedural failures, must be monitored separately.

In general, the articles with data most appropriate for a benchmark orientation have demonstrated that robotic assistance generally increases technical accuracy and possibly improves select patient-centred or early recovery outcomes, such as satisfaction, MCIDs, and early range of motion restoration, while not consistently improving joint awareness, range of motion, neuromotor function, or complication profiles at 1–2-year follow-up. For this reason, not just mean difference in study outcomes but individual patients’ outcomes compared to their own preoperative status and to benchmark outcomes must also be considered when judging robotic TKA; consequently, preoperative data, absolute outcome data, MCIDs or patient acceptable symptom state, satisfaction, rate of manipulation under anaesthesia, readmission, infection, and robotic-specific complications are all potential outcome measures. Ultimately, such an approach shifts from evaluation of “is robot better than manual” to “can robots help us achieve the benchmark outcome of excellent TKA care?”

## 9. Review Limitations and Future Directions

Although robotic system types and alignment strategies were extracted and reported where available, these variables were treated descriptively rather than as the basis for subgroup analysis. This approach was chosen because the primary objective of this review was to evaluate the overall influence of robotic assistance on postoperative outcomes after TKA rather than to compare specific robotic platforms, imaging workflows, or alignment philosophies. Moreover, the available literature remains heterogeneous, with differences in robot design, imaging strategy, implant type, surgeon experience, alignment targets, and rehabilitation protocols. In several studies, alignment strategy was not explicitly reported or differed between rTKA and mTKA groups, which may act as a confounding factor when interpreting outcomes. Therefore, while robot type and alignment philosophy may partly explain variability in PROMs, ROM, neuromotor recovery, and complications, the current evidence does not allow reliable conclusions regarding the superiority of one robotic system or alignment approach over another.

Another important limitation of this review is that it was designed as a narrative review and not as a systematic review or meta-analysis. Consequently, the search strategy did not follow a predefined protocol for the literature’s retrieval, inclusion, and bias assessment, as required by PRISMA guidelines. While this format allows a broader synthesis of current evidence and the reasons behind these results, it also introduces potential selection bias, since study inclusion depended partly on the authors judgement and data accessibility. Therefore, the conclusions presented should be interpreted as a qualitative integration of current trends and evidence gaps rather than as definitive comparative efficacy statements.

Looking ahead, there are several directions for future research. High-quality randomised controlled trials and large multicentre cohorts with standardised methodologies are essential to validate early advantages observed with robotic assistance and to determine their durability over long-term follow-up. Consistent reporting of alignment strategies, robotic system characteristics, implant design, and rehabilitation protocols will contribute to more meaningful comparisons across studies. Additionally, greater integration of neuromotor metrics, such as proprioception testing, advanced gait analysis, and electromyography, may help explain how robotic precision contributes to early recovery. Emerging artificial intelligence technologies may further support this evolution by enhancing preoperative planning, enabling intraoperative decision support, and facilitating more detailed postoperative functional assessment through wearable sensors and gait analytics. However, these applications remain largely investigational and require further validation before routine clinical implementation [100,101].

## 10. Conclusions

Current evidence shows that while rTKA does not consistently produce notable superior results in PROMs, ROM, neuromotor recovery, or complication rates, the technique demonstrates small but reproducible early benefits in selected domains, including joint awareness, balance, gait symmetry, quadriceps activation, and patient satisfaction. Importantly, even if the gains at the individual level are small, they may translate into substantial population-level impact when viewed in the context of the rapidly increasing global burden of knee osteoarthritis and the rising volume of TKA procedures. Earlier functional recovery, improved mobility, and faster normalisation of gait not only enhance patient quality of life but also reduce length of hospital stay, accelerate return to independence, and may lower cumulative healthcare expenditures. Thus, although the clinical differences between rTKA and mTKA may appear subtle in isolated metrics, the aggregated benefits of greater precision and more predictable recovery trajectories could hold significant value for patients, healthcare systems, and society at large. Continued large-scale, high-quality research will be essential to clarify whether these early advantages translate into meaningful long-term improvements and cost savings.

## Figures and Tables

**Table 1:** Summary of representative studies reporting PROMs after rTKA versus mTKA.

Study	Study Population	PROMs Evaluated	Results	Robot	Alignment
Geng et al. [45]	65 patients in rTKA and 65 patients in mTKA	KSS, WOMAC, and SF-36 at 6 weeks postoperatively	No statistically significant differences in measured PROMs between groups	AKEC (CT-based, semi-active)	Mechanical
Naziri et al. [46]	40 patients in rTKA group and 40 patients in mTKA group	KSS and LEAS at 30, 60, and 90 days postoperatively	No differences in KSS or LEAS at any time interval	MAKO (CT-based, semi-active)	Mechanical
Golinelli et al. [47]	161 patients in mTKA group and 214 patients in rTKA group	EQ-VAS, KOOS-PS, and EQ-5D at 6 months postoperatively	Significant improvements in all measured PROMs in rTKA group, except for KOOS-PS where there were no differences	MAKO (CT-based, semi-active)	Functional (rTKA) vs. mechanical (mTKA)
Han et al. [48]	29 patients in rTKA group and 32 patients in mTKA	KSS and WOMAC at 6 months postoperatively	Improved KSS in rTKA group	CUVIS Joint (CT-based, active)	Mechanical
Albelooshi et al. [49]	117 patients in rTKA group and 34 patients in mTKA group	KOOS-JR, VR-12 (mental and physical), and KSS satisfaction at 3, 6, 12, and 24 months postoperatively	No significant differences in measured PROMs between the two groups	NAVIO (imageless, semi-active)	Constitutional (~3° varus) in rTKA vs. mechanical in mTKA
Mitchell et al. [50]	140 patients in rTKA group and 132 patients in mTKA group	SF-12 (mental and physical), VR-12 (mental and physical), KOOS-JR, and UCLA at 1 year postoperatively	No differences in measured PROMs	MAKO (CT-based, semi-active)	Mechanical
Yamamoto et al. [51]	53 patients in rTKA group and 41 patients in mTKA group	KSS (symptom, patient satisfaction, patient expectation, and advanced activities) and FJS-12 at 1 and 2 years postoperatively	No differences in KSS subscales, but FJS-12 was higher in mTKA group	NAVIO (imageless, semi-active)	Mechanical
Liow et al. [52]	31 patients in rTKA and 29 patients in mTKA group	KSS, OKS, subscales of SF-36, and patient satisfaction (measured by a 6-point Likert scale) at 6 and 24 months postoperatively	No differences in OKS or KSS, but rTKA was associated with higher scores of SF-36 vitality and emotional role. No differences in satisfaction rate over the 2-year follow-up period	ROBODOC (CT-based, active)	Mechanical
Held et al. [53]	111 patients in rTKA group and 110 patients in mTKA group	KSS-FS, WOMAC, and SF-12 (mental and physical) at 3, 12, and 24 months postoperatively	No differences at any time in measured PROMs	NAVIO (imageless, semi-active)	Mechanical
Stoltz et al. [54]	393 patients in rTKA group and 312 patients in mTKA group	KSS, FJS-12, WOMAC, KOOS-JR, and patient satisfaction (measured by a 5-point Likert scale) at 2 years postoperatively	Higher KSS, WOMAC, and KOOS-JR scores in rTKA patients. No differences in FJS-12. Increased satisfaction rate after rTKA; differences were statistically significant.	CT-based, semi-active (brand not specified)	Functional (rTKA) vs. mechanical (mTKA)
Eerens et al. [55]	73 patients in rTKA group and 74 patients in mTKA group	OKS, FJS-12, and EQ-5D at 2 years postoperatively	Higher FJS-12 and OKS in rTKA group with no differences in EQ-5D	NAVIO (imageless, semi-active)	Kinematic (rTKA) vs. mechanical (mTKA)

**Table 2:** Summary of representative studies reporting ROM after rTKA versus mTKA.

Study	Study Population	Postoperative Follow-Up	Results	Robot	Alignment
Major et al. [67]	729 patients in mTKA group and 395 patients in rTKA group	2 and 6 weeks	No differences in ROM between groups	CORI (imageless, semi-active)	Mechanical (mTKA) vs. functional (rTKA)
Fary et al. [68]	216 patients in mTKA and 216 patients in rTKA group	1 and 3 months	Less loss of ROM at 1 month in rTKA group and faster recovery (achieving 90° of flexion) at 3 months postoperatively	ROSA (imageless, semi-active)	Not standardised
Naziri et al. [46]	40 patients in rTKA group and 40 patients in mTKA group	30, 60, and 90 days	Improved ROM in rTKA group at 90 days compared to mTKA	MAKO (CT-based, semi-active)	Mechanical
Tian et al. [69]	72 patients in rTKA group and 72 patients in mTKA	3 months	No differences in ROM between groups	JIANJIA (CT-based, semi-active)	Mechanical
Xu et al. [70]	37 patients in rTKA group and 35 patients in mTKA group	90 days	No differences in ROM between groups	YUANHUA (CT-based, semi-active)	Mechanical
Han et al. [48]	29 patients in rTKA group and 32 patients in mTKA	6 months	Greater improvement in ROM after rTKA	CUVIS Joint (CT-based, active)	Mechanical
Held et al. [53]	111 patients in rTKA group and 110 in mTKA group	3, 12, and 24 months	No differences in ROM between groups	NAVIO (imageless, semi-active)	Mechanical
Liow et al. [52]	31 patients in rTKA group and 29 patients in mTKA group	2 years	No differences in ROM between groups	ROBODOC (CT-based, active)	Mechanical
Yamamoto et al. [51]	53 patients in rTKA group and 41 patients in mTKA group	1 and 2 years	Slight improvement in ROM in rTKA group with better extension but not statistically significant	NAVIO (imageless, semi-active)	Mechanical
Stoltz et al. [54]	393 patients in rTKA group and 312 in mTKA group	2 years	Extension outcomes were comparable between groups; the small but statistically significant difference in flexion did not reach clinical importance	CT-based, semi-active (brand not specified)	Functional (rTKA) vs. mechanical (mTKA)

Note: All studies reported preoperative/baseline ROM assessment unless otherwise stated; therefore, the table reports only postoperative follow-up time points.

**Table 3:** Summary of representative studies reporting neuromotor recovery after rTKA.

Study	Study Population	Neuromotor Assessments	Results	Robot	Alignment
Sanz et al. [74]	101 patients in rTKA group	Quadriceps muscle activation in static and dynamic conditions at 4 weeks postoperatively	Lower activation on the operated limb compared with non-operated	MAKO (CT-based, semi-active)	Not reported
He et al. [80]	31 patients in rTKA group and a control group (consisting of the contralateral limb of the same patient, with no interventions)	3D gait analysis with spatiotemporal, kinematic, and kinetic parameters recorded before and at 3 and 6 months postoperatively	3D gait analysis showed reduced double-support time and greater knee motion after rTKA; minor residual asymmetry persisted versus the contralateral limb	SkyWalker (CT-based, semi-active)	Mechanical
Han et al. [48]	29 patients in rTKA group and 32 patients in mTKA group	Isokinetic muscle force using a dynamometer at 6 months postoperatively	Small improvements in favour of rTKA regarding isokinetic muscle force, but not significant	CUVIS Joint (CT-based, active)	Mechanical
Ajekigbe et al. [81]	50 patients in rTKA group and 50 patients in mTKA group	Gait cycle parameters, anteroposterior and lateral sway, and plantar pressure ratios before and at 1 year postoperatively	Reduced propulsion time in rTKA; shorter foot-flat and mid-stance phases in mTKA; lateral sway decreased in rTKA; hindfoot loading increased postoperatively in both groups with no between-group difference	MAKO (CT-based, semi-active)	Mechanical
Bayrak et al. [82]	25 patients in rTKA group and 25 patients in mTKA group	Berg Balance Scale (BBS) and Lysholm Knee Scoring Scale at 18 months postoperatively	Statistically significant improvement in balance performance on the BBS in rTKA group, but no differences in Lysholm Scale	CORI (imageless, semi-active)	Mechanical in mTKA, not reported in rTKA

**Table 4:** Summary of representative studies reporting complications after rTKA.

Study	Study Population	Follow-Up Period	Results	Robot	Alignment
Kayani et al. [94]	60 patients in rTKA and 60 patients in mTKA group	>1 month	One wound complication in each group; in rTKA, wound dehiscence related to registration pins, treated with regular dressings and prophylactic antibiotics.	MAKO (CT-based, semi-active)	Mechanical
Naziri et al. [46]	40 patients in rTKA group and 40 patients in mTKA group	1, 2, and 3 months	One complication in mTKA (arthrofibrosis requiring manipulation under anaesthesia)	MAKO (CT-based, semi-active)	Mechanical
Smith et al. [95]	120 patients in rTKA group and 103 patients in mTKA	>1 year	17 complications reported in rTKA group and 13 in mTKA group; in both groups, nine cases required manipulation under anaesthesia	MAKO (CT-based, semi-active)	Mechanical
Mahure et al. [96]	115 patients in rTKA group	>1 year	No complications related to rTKA	TSolution One (CT-based, active)	Mechanical
Mitchell et al. [50]	140 patients in rTKA group and 132 patients in mTKA group	>1 year	No significant differences in complications between groups	MAKO (CT-based, semi-active)	Mechanical
Liow et al. [52]	31 patients in rTKA group and 29 patients in mTKA group	>2 years	Seven complications observed (5 in rTKA group and 2 in mTKA); only two complications required reintervention (both in rTKA group—periprosthetic joint infection and tibial malpositioning with persistent lateral-sided knee pain)	ROBODOC (CT-based, active)	Mechanical
Held et al. [53]	111 patients in rTKA group and 110 patients in mTKA group	>2 years	No statistical differences in complications between groups; five reinterventions required in mTKA and six in rTKA group; one tibial shaft non-displaced fracture attributed to pin site in rTKA group	NAVIO (imageless, semi-active)	Mechanical
Albelooshi et al. [49]	117 patients in rTKA group and 34 patients in mTKA group	>2 years	One femoral component revision for periprosthetic fracture in rTKA group	NAVIO (imageless, semi-active)	Constitutional (~3° varus) in rTKA vs. mechanical in mTKA
Stoltz et al. [54]	393 patients in rTKA group and 312 patients in mTKA group	>2 years	No significant differences in complications between groups	CT-based, semi-active (brand not specified)	Functional (rTKA) vs. mechanical (mTKA)
Song et al. [97]	50 patients in rTKA group and 50 patients in mTKA group	>41 months	No difference in the complication rates between the two groups; all were successfully treated using nonsurgical treatment	ROBODOC (CT-based, active)	Mechanical

## Data Availability

No new data were generated during this study.

## Abbreviations

The following abbreviations are used in this manuscript:

aHKA	Arithmetic Hip–Knee–Ankle Angle
CT	Computed Tomography
EMG	Electromyography
EQ-5D	EuroQol 5-Dimension Questionnaire
EQ-VAS	EuroQol Visual Analogue Scale
FJS-12	Forgotten Joint Score-12
KA	Kinematic Alignment
KSS	Knee Society Score
LOS	Length of Stay
MA	Mechanical Alignment
MCID	Minimal Clinically Important Difference
MRI	Magnetic Resonance Imaging
mTKA	Manual Total Knee Arthroplasty
OKS	Oxford Knee Score
PROM	Patient-Reported Outcome Measure
QoL	Quality of Life
ROM	Range of Motion
rTKA	Robotic-Assisted Total Knee Arthroplasty
SF-12	Short Form-12 Health Survey
SF-36	Short Form-36 Health Survey
TKA	Total Knee Arthroplasty
TUG	Timed Up and Go test
UKA	Unicompartmental Knee Arthroplasty
VR-12	Veterans RAND 12-Item Health Survey
WOMAC	Western Ontario and McMaster Universities Osteoarthritis Index
6MWT	Six-Minute Walk Test

## Appendix A

Oxford Knee Score (OKS)—The Oxford Knee Score is a knee-specific PROM for patients undergoing total knee arthroplasty. It consists of 12 questions evaluating pain, functional ability, and difficulty performing daily activities over the preceding four weeks. It provides a straightforward, patient-centred assessment of postoperative recovery [102].

Knee Society Score (KSS)—The Knee Society Score is a combined clinician- and patient-reported instrument assessing pain, stability, knee range of motion, and functional capacity. Modern versions include additional subscales for patient satisfaction, expectations, and advanced activities, offering a comprehensive evaluation of clinical and functional outcomes after TKA [103].

Western Ontario and McMaster Universities Osteoarthritis Index (WOMAC)—WOMAC is a validated PROM widely used in osteoarthritis and joint replacement research. It includes three subscales—pain, stiffness, and physical function—measuring symptoms experienced during daily activities. It is sensitive to clinical change and commonly used to track postoperative improvement after TKA [104].

Knee Injury and Osteoarthritis Outcome Score (KOOS/KOOS-JR)—The KOOS assesses knee pain, symptoms, function in daily living, function in sports, and knee-related quality of life. KOOS-JR is an abbreviated 7-item version optimised for arthroplasty patients. Both instruments quantify functional recovery and residual symptoms following TKA [105].

Forgotten Joint Score-12 (FJS-12)—The Forgotten Joint Score evaluates joint awareness during daily activities. Lower scores reflect greater awareness of the artificial knee, whereas higher scores indicate a more “natural” knee feel. It is particularly sensitive at detecting mid-flexion instability or subtle functional differences between surgical techniques [106].

Short Form Health Surveys (SF-12/SF-36)—SF-12 and SF-36 are generic health-related quality of life measures assessing physical, emotional, and social well-being. They provide broad information beyond joint-specific function, including vitality, mental health, physical limitations, and overall health perception [107].

EuroQol-5 Dimensions (EQ-5D) and EQ-VAS—The EQ-5D evaluates mobility, self-care, usual activities, pain/discomfort, and anxiety/depression, generating a standardised health utility index. The accompanying EQ-VAS records the patient’s self-rated health on a 0–100 scale. These measures reflect overall well-being and quality of life rather than knee-specific function [108].

UCLA Activity Score—The UCLA scale measures postoperative activity level by rating participation in physical activities from sedentary behaviour to high-impact sports. It reflects the patient’s functional aspirations and return to desired activity levels after TKA [109].

Veterans RAND 12-Item Health Survey (VR-12)—The VR-12 assesses physical and mental health status across eight domains, including emotional well-being, social functioning, pain, and general health perception. Similar to SF-12, it provides a broad overview of health-related quality of life [110].

Berg Balance Scale (BBS)—The Berg Balance Scale is a performance-based assessment used to evaluate static and dynamic balance during functional tasks. It includes 14 standardised activities—such as standing, reaching, turning, and transferring—each scored from 0 to 4. The total score reflects the patient’s balance control and fall risk. Although not knee-specific, it is frequently used in TKA research to assess postoperative proprioception, postural stability, and neuromotor recovery [85].

Lysholm Knee Scoring Scale—The Lysholm Knee Scoring Scale is a knee-specific PROM designed to evaluate symptoms and functional limitations related to instability, pain, locking, swelling, stair climbing, and limp. Originally developed for ligament injuries, it is also used following TKA to assess patient-perceived knee function and activity tolerance. Scores range from 0 to 100, with higher scores indicating better knee performance [111].
